# The 100-Days: Physical Exercise and Challenges to Assess, Maintain and Improve Physical Fitness During Lockdown

**DOI:** 10.3390/sports12120337

**Published:** 2024-12-05

**Authors:** Tommaso Di Libero, Annalisa D’Ermo, Beatrice Tosti, Stefano Corrado, Pierluigi Diotaiuti, Angelo Rodio

**Affiliations:** Sustainable Living Concept Laboratory “Marco Marchetti” (Xlab), Department of Human, Social and Health Sciences, University of Cassino and Southern Lazio, Via S. Angelo, Campus Folcara, 03043 Cassino, FR, Italy; tommaso.dilibero@unicas.it (T.D.L.); beatrice.tosti@unicas.it (B.T.); stefano.corrado@unicas.it (S.C.); p.diotaiuti@unicas.it (P.D.); a.rodio@unicas.it (A.R.)

**Keywords:** COVID-19, physical activity, fitness level, functional assessment, remote testing, challenge

## Abstract

The COVID-19 pandemic reduced physical activity and increased sedentary behavior, raising health risks. To combat this, a 100-day training program was designed to maintain and improve fitness during lockdown. This program, which included a challenge with physical assessments and online sessions, aimed to enhance fitness and motivation. Methods: Twenty participants (mean age 45.2 ± 12.7 years) with good baseline fitness completed strength, endurance, coordination, and flexibility exercises over 100 days, with monthly challenges. Fitness was assessed at baseline (T0), mid-program (T1), and completion (T2). Results: Males initially showed higher BMI and mild cardiovascular risks. Flexibility improved for both genders, with females increasing from 12.5 ± 4.51 cm to 14.8 ± 6.65 cm and males from 4.4 ± 6.33 cm to 8.8 ± 10.69 cm. Males’ vertical jump height increased from 20.7 ± 13.05 cm to 28.2 ± 10.49 cm. In the challenge, push-up repetitions rose for both genders, achieving excellent scores (>24 reps for females, >25 for males). Males advanced in the repeated crunch test, while females consistently excelled in the repeated squat. Weight and BMI reductions were also observed, particularly in overweight males. Conclusions: The 100-day training program, combined with the challenge, effectively sustained physical fitness and motivation among participants during pandemic-related restrictions. Notable strength and endurance improvements were observed across both genders, reinforcing the potential of interactive, remote training programs to promote physical health in periods of limited activity.

## 1. Introduction

During the COVID-19 pandemic, declared by the World Health Organisation (WHO) in 2019, the world population found itself in prolonged isolation, necessary to contain the spread of the virus [1]. This condition has led to a drastic reduction in social interactions and an inability to regularly engage in outdoor activities, including recreational and agonistic exercise [2]. One of the most significant impacts has been the substantial increase in time spent in sedentary activities, particularly prolonged screen exposure [3]. This shift exacerbated the negative effects of unhealthy lifestyle, amplifying the effect induced by hypokinesis, known to be linked to an increased risk of developing non-communicable diseases [4,5]. These negative effects also extend to individuals who previously engaged in regular physical activity, compromising their lifestyle and general well-being [6]. Although the Italian government permitted individual outdoor exercise, only areas near home could be used to practice physical activity to avoid gatherings. As a result, many people, particularly those living in urban settings, were forced to train at home, often resorting to digital solutions such as sparring apps. These activities, however, were self-managed and lacked supervision from personal trainers [7]. Consequently, during and overall after the pandemic, attention was focused on preventive actions that various governments could take to encourage the population to engage in physical activity, a strategic preventive health action also highlighted as an objective in the UN 2030 Agenda to reduce governmental healthcare costs [8]. Remote fitness interventions emerged as a critical alternative to address the challenges of restricted mobility and limited access to traditional fitness resources. These included online training programs, live-streamed fitness classes, and mobile applications designed to support physical activity at home. While such solutions provided a valuable means to maintain physical activity levels, they also introduced a set of challenges. Limited access to necessary equipment, varying levels of digital literacy, and a lack of personalized guidance were significant barriers to their effectiveness [9,10]. Moreover, maintaining engagement and motivation in a virtual setting proved challenging for many individuals, as the absence of social interaction and external accountability often led to reduced adherence [11,12,13]. Despite these challenges, remote fitness interventions achieved notable outcomes in specific populations, demonstrating their potential to partially mitigate the negative impacts of reduced physical activity during the pandemic. Studies highlight that these interventions contributed to preserving mental well-being, reducing stress, and maintaining a baseline level of physical fitness for individuals who actively participated in them [14,15,16]. In light of these findings, attention during and after the pandemic has focused on preventive actions governments and organizations could take to encourage populations to engage in physical activity. This aligns with strategic health objectives highlighted in the UN 2030 Agenda to reduce governmental healthcare costs [8]. Regarding the goals mentioned in the Agenda, several studies have investigated the relationship between fitness levels, a normal body weight, and the risk of developing severe symptoms related to COVID-19. In particular, a higher aerobic capacity may reduce the likelihood of experiencing severe symptoms and being hospitalized in an intensive care unit [17,18,19]. In Italy, during the lockdown, the part of the population that practiced motor activity continued to maintain, or even increase, the time spent exercising, thus remaining active. In contrast, those with less or no physical activity spent the lockdown period without exercise [20,21]. Indeed, these subjects were mainly related to daily commuting, such as going to work, school, or grocery shopping, and even the slightest movement was suspended during the lockdown. Consequently, they spent the lockdown period in total inactivity, further amplifying the adverse effects of sedentary behavior [22,23].

In addition to physical inactivity, the imbalance between caloric intake and energy expenditure has worsened, leading to a rise in the number of overweight and obese individuals since the onset of the COVID-19 pandemic [24]. Moreover, the ability of active individuals to maintain physical activity during the pandemic was influenced by a range of factors, including their pre-pandemic activity levels, access to home workout equipment, social support, income, personal lifestyle habits, and contextual elements such as available space and online training options. This highlights the complexity of maintaining training volumes and intensities, particularly for individuals accustomed to moderate to high levels of physical activity or specific sports [25,26,27]. Web platform use became essential to continue exercising through instructor-led sessions, often via videos posted on different platforms. However, while representing a valid alternative, these digital activities had certain limitations. In particular, the lack of personalized feedback and adaptation to individual fitness levels increased the risk of injury and reduced training effectiveness without proper supervision [28,29]. Furthermore, the absence of social interactions during exercise negatively affected people’s motivation, leading to a high drop-out rate and a further increase in sedentary behavior [30,31,32,33]. To address these challenges, efforts were made to develop strategies that could encourage physical activity during the lockdown, focusing on the potential of digital tools to provide more interactive, adaptive, and motivational support. These approaches aimed to engage inactive and active populations in maintaining a healthy lifestyle despite restrictive conditions [34,35]. One effective approach was administering training programs considering each individual’s fitness status, thus minimizing the risk of injury and activity drop-out. To this end, a 100-day program was to assess by mean evaluation test and maintain or improve fitness levels through adapted and monitored training sessions. To better engage participants and increase their motivation, periodic challenges focused on physical activity and goal achievement were included to sustain motivation throughout the program. These challenges provided benchmarks for progress and kept participants engaged over time. In addition, all interactions between instructors and participants were structured to ensure proper exercise execution, adhering to established training principles such as frequency, duration, volume, and intensity. The use of a digital platform facilitated real-time guidance, corrections, and feedback, creating an interactive and supportive environment. The hypothesis of this approach was aimed at improving performance and making physical activity an enjoyable and healthy experience. By integrating these elements, the program maintained participants’ engagement and motivation despite the constraints of the block. Over time, all interactions between instructors and users aim to guide performance based on training frequency, duration, volume, and intensity. Through the digital platform, these interactions enabled real-time corrections, feedback, and encouragement, helping to improve performance, make physical activity an enjoyable experience, and promote overall health and well-being [36,37]. In addition, periodic challenges were administered to assess and maintain high motivation levels and avoid drop-out.

## 2. Materials and Methods

### 2.1. Participants

In this study, 20 participants (10 females and 10 males) were enrolled, all of whom already had a medical certificate for healthy sports activities and were confirmed to be COVID-free. A conditionof participation in the programme was to maintain a healthy diet throughout the duration of the protocol. The participants were selected from among students, administrative and teaching staff from the University of Cassino. Informed consent and authorization about benefits and risks were obtained in accordance with the Declaration of Helsinki for Human Research of 1964. This work was approved by the Institutional Review Board of the University of Cassino and Southern Lazio (no. 24777.2022.12.12).

### 2.2. Protocol

**Figure 1:**
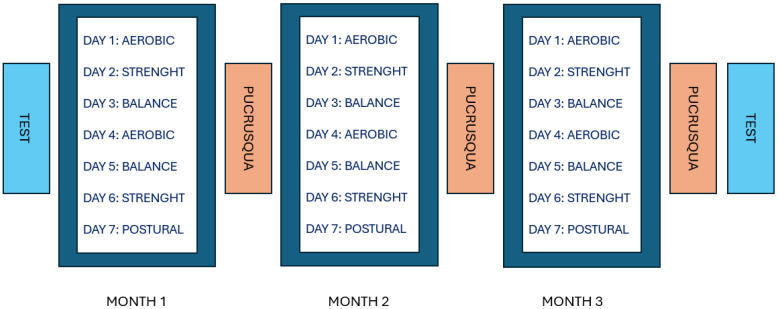
The three months program is described in this image. We indicated as TEST the battery test used to evaluate the sample. This battery was performed at the beginning and the end of the program; as PUCRUSQUA, we indicated the three motor tasks: push up, crunch and squat; the PUCRUSQUA was conducted at the end of each month; the 7 days program indicated, was repeated for each week of the three months; PUCRUSQUA was conducted on a separate day, every 15th of the month, the same for the last test session.

Participants measured their weight with a personal scale and height with a tailor’s tape, following instructions using Google Meet (2020, Google LLC., Web version, Mountain View, CA, USA) web platform for video call. They were guided to stand barefoot against a wall, mark the top of their head with a pencil and measure the distance from the floor to the mark with a tape measure. Following the operators’ instructions, subjects measured abdominal circumference measures, which were used to identify the risk of cardiovascular disease based on reference tables [38,39]. Anagraphic and anthropometric data were collected remotely and are shown in Table 1. To characterize the fitness level of each subject and define the appropriate workloads, all participants underwent tests to assess their coordinative and conditional abilities, always carried out remotely under an operator’s supervision. The remote testing methods used have been validated by a previous study that assessed repeatability, validity and concordance against the same tests carried out in presence. [35,40]. The battery test consisted of: Push-Up (PUp) [41]; Squat Jump (SJ) [42]; Ruler test (RT) [43], right hand (RTR) and left hand (RTL); Stork test (ST) [44], right leg (STR) and left leg (STL); V-Sit & Reach (V-S&R) [45]; Ruffier’s test [46]. Tests were reconducted at the end of the third month to monitor the effect induced by the training program. Following the initial assessment, physical activity sessions were conducted, including activities to improve conditional and coordinative abilities, such as aerobic circuits, strength-focused training, proprioception and balance exercises, postural exercises, and high-intensity circuits, named the 100-days program, as shown in Figure 1. The study duration was explicitly chosen to coincide with the restrictions imposed by the government during the COVID-19 pandemic and the subsequent lockdown phases. This period included the strict lockdown phase (Phase 1, from 3 March to 3 May, lasting 60 days), followed by a transition phase (Phases 2 and 3, from 4 May to 14 June). This structure allowed participants to adhere to the prescribed exercise regimen under certain conditions, enabling a comprehensive mid-term evaluation of fitness adaptations. Beyond government regulations, in order to complete the programme and allow for the final functional evaluations and the last challenge, in context and total sum, the programme lasted 100 days. The 100-days training program consisted of activities spread over 7 days a week, divided as follows: 2 days for power exercises, 2 days for endurance exercises, 2 days focused on coordination and mobility, and 1 day reserved for postural activities, as shown in Table 1. The training program, mainly based on calisthenics exercises, was adapted to individual fitness level obtained in the battery test, facilitating continuity and preventing the risk of injuries and drop-out. The training was tailored by varying the workload through different weights (½l, 1l, 2l and 5l bottles) or modulating the frequency of execution of the motor task, exercise time and recovery time. Moreover, the PUCRUSQUA challenge, an acronym for Push-ups, Crunches, and Squats, was implemented over a three-month period. These exercises were chosen not only for their simplicity and accessibility but also for their ability to engage the cardiovascular system and their safety in confined spaces, making them particularly suitable for home workouts during lockdown conditions. Specifically, two high-intensity exercises were included due to their significant cardiovascular demands, with MET values ranging from 8 to 10 [47]. This approach balanced rigorous physical performance assessment and participant safety while maintaining engagement throughout the program. This challenge was repeated three time over the 100 days on 15 April, 15 May, and 15 June. The PUCRUSQUA, besides being considered a practical training session, involved the participants dynamically, stimulating not only competition but also cooperation and social interaction, keeping commitment and motivation high [48]. After a five-minute warm-up with calisthenic exercises, participants must perform a series of push-ups (R_Push-Up_), repeated crunches (R_Crunch_) and squats (RS) to exhaustion. Five minutes of active cool-down were included between each trial, concluding the challenge with a final goal of 180 s of Quick Feet Squatting (QFS). The challenge, including warm-up and cool-down, lasted approximately 35 min. At the end of each training session or challenge, stretching exercises were always performed to aid muscle recovery and improve flexibility [49].

## 3. Statistical Analysis

Descriptive statistics were performed to characterize the sample, with means and standard deviations calculated for both anthropometric data, assessment test and challenge results across all participants, as presented in Table 2, Table 3 and Table 4. Shapiro-Wilk normality tests were applied to each variable to examine the distributional properties, allowing for comparisons between male and female participants. When the assumption of normality was met across assessment time points (T0, T1, and T2), a one-way analysis of variance (ANOVA) was conducted to identify statistically significant differences within the data, marked by an asterisk in the tables. For ANOVA results that were statistically significant (p≤0.05), post hoc comparisons were subsequently performed to specific pairwise group differences.

## 4. Results

Table 1 shows the participants’ anagraphich and anthropometric data. All data were normally distributed, with the Shapiro-Wilk test, resulting for females, age (*p* = 0.137), height (*p* = 0.662), male age (*p* = 0.108), height (*p* = 0.743), body mass (BM, *p* = 0.391), or BMI (*p* = 0.737). Concerning participants’ BMI measurements indicated a normal weight for females (22.6 ± 2.08 kg/m^2^) and a slightly overweight value for males (25.2 ± 3.28 kg/m^2^). The latter was mainly due to the presence of three overweight subjects. Regarding indirect information on the risk of cardiovascular disease obtained through abdominal circumferences, females averaged 80 cm and males 88 cm, respectively (low-risk, Female 70–89 cm; male 80–99 cm). The same three subjects also exhibited elevated abdominal circumference values, suggesting an increased risk of dysmetabolic and cardiovascular diseases. Additionally, they met two of the three metabolic syndrome criteria. In the flexibility test (V-S&R), females showed values of 12.5 ± 4.51 cm at T0 and 14.8 ± 6.65 cm at T1, while males 4.4 ± 6.33 cm at T0 and 8.8 ± 10.69 cm at T1, indicating for both well above average values (12–14 cm for females and 7–11 cm for males). In the RTR and RTL tests, females showed values of 18.1 ± 1.37 cm at T0 and 19.5 ± 1.97 cm at T1 for the right hand (70–200 ms, good value) and 10.5 ± 2.40 cm at T0 and 12.4 ± 5.95 cm at T1 for the left hand (<190 ms, excellent value). Males displayed values of 33.6 ± 7.34 cm at T0 and 34.7 ± 3.09 cm at T1 for the right hand (270–300 ms, low value) and 23.7 ± 2.68 cm at T0 and 22.4 ± 2.86 cm at T1 for the left hand (270–300 ms, average value). Both genders displayed low values (<10 s) in all conditions at T0 and T1 for the STR and STL balance tests. In the SJ test, females achieved a vertical jump displacement of 22.8 ± 5.65 cm at T0 and 23.0 ± 6.68 cm at T1, indicating a very good value (range 20–24 cm). On the other hand, for males, the displacement increased from 20.7 ± 13.05 cm at T0 to 28.2 ± 10.49 cm at T1, indicating an amelioration from the good (20–24 cm) to the very good value (24–28 cm). In the PUp, both males and females sustained excellent values (female, >24 rep; male, >25 rep) during both phases (T0 and T1). In the Ruffier test, females showed values of 6.5 ± 2.14 at T0 and 9.8 ± 2.90 at T1, while males 7.7 ± 4.41 at T0 and 8.5 ± 4.61 at T1, indicating low (Ruffier index 6–8) or very low (Ruffier index 8–10) values in aerobic endurance for both. Regarding the PUCRUSQUA, the number of repetitions in the R_Push-Up_ improved for both females and males between T0 and T2, consistently classified as excellent value in all three conditions (females > 24 rep; males > 25 rep). For the R_Crunch_, as shown in Table 3, females obtained good values at T0 and T1 (70–80 rep), while at T2, resulting in an average value (50–60 rep). Males showed average values at T0 but achieved excellent values at T1 and T2 (<90 rep). For the R_Squat_ and QFS tests, no comparison tables are available, as these activities were included to make the challenge more engaging by incorporating familiar motor tasks like the PUp and SJ. In the R_Squat_ test, both females and males showed increased repetitions from T0 to T2, with females consistently achieving higher values across all three sessions than males. Regarding the QFS test, the target of 180 s was not reached at T0 by either females (150 ± 21.6 s) or males (153 ± 26.9 s). However, both genders successfully met the target at T1 and T2. Regarding anthropometric data, only males showed a decrease in weight and BMI after 100 training days, with a marked weight loss observed in the three overweight participants. Regarding test results at T0 and T1, only females showed statistically significant differences in flexibility and balance, with an improvement in flexibility and a decrease in balance. Males showed a similar trend. Regarding the PUCRUSQUA results, females and males showed statistically significant improvements from T0 to T1 and T0 to T2, except in the R_Crunch_ for females. No statistically significant differences were observed in any of the PUCRUSQUA exercise tests between T1 and T2.

## 5. Discussion

The COVID-19 pandemic significantly increased sedentariness and reduced regular physical activity, creating significant challenges for overall wellness and health. The 100-day program, coupled with the PUCRUSQUA, was designed to respond to these problems by providing structured physical activity to perform at home that could support and improve the participants’ fitness during lockdown. The results suggest that although home training has limitations, a well-designed remote program can help maintain a sufficient fitness level. A statistically significant difference was observed when comparing the results of push-ups performed during the PUCRUSQUA with those from evaluation tests. Specifically, the push-ups conducted in the challenge yielded superior results than those obtained in standard evaluation tests.

About the evaluation test, the participants performed the motor task to the best of their ability. However, the introduction of a challenge component had a significant impact on the results obtained in the PUCRUSQUA. This improvement suggests that the motivation from the challenge enhanced the participants’ commitment, leading them to exceed their usual performance levels, even though they were performing the same motor task. In other words, the challenge dimension appears to have activated additional psychomotor resources, enabling the subjects to perform better than they did in the standard test, although the task performance conditions remained unchanged [50]. Since PUCRUSQUA consists of a sequence of exercises involving both the upper and lower limbs and the core, we can state, in light of the previous discussion, that it can be used as a functional assessment method, and further analysis of its specific features contributing to these outcomes may provide valuable insights for optimizing both evaluation and training practices. In addition to the physical benefits, it is possible to speculate that the 100-days program also contributed to the participants’ mental well-being [51,52], possibly due to physical activity’s role in reducing stress and improving mood by releasing endorphins [53]. Following a structured routine and participating in monthly challenges may have created a sense of commitment and motivation, two elements known to counteract the adverse effects of social isolation [54]. Moreover, bi-directional online interaction between the user and trainer allowed feedback and helped maintain correct technique during the exercises, reducing the risk of injury and possible drop-out [55].

The results indicate a general improvement in strength and endurance, which is a potentially critical factor in reducing the risk of hospitalization, especially in populations at higher risk of severe illness due to conditions like obesity, diabetes, and cardiovascular diseases [56,57,58,59]. This improvement aligns with findings from previous studies that have shown the benefits of regular physical activity in enhancing both muscular strength and cardiovascular health, thereby improving overall resilience against illness [60,61].

However, while the program showed positive results in strength and endurance, the aerobic activities included were not intense enough to sufficiently engage aerobic metabolism, particularly beyond the ventilatory threshold [62]. Specifically, the maximum duration of certain activities, such as the 180-s duration for the QFS, and the total session length of 60 min, were limiting factors in achieving the necessary intensity for sustained aerobic conditioning. This means that the program, while beneficial for overall health, might not be sufficient to fully replace more traditional, higher-intensity aerobic exercises like running, cycling, or swimming, which are known to engage the cardiovascular system over extended periods and at higher intensities [63,64]. Additionally, the absence of specific equipment and adequate space in participants’ environments likely restricted the variety and intensity of exercises that could be performed, further limiting the program’s effectiveness for improving aerobic capacity. While some adaptive strategies were employed, such as using water bottles of various sizes as makeshift weights, these alternatives cannot fully replicate the resistance offered by traditional weights or machines, nor can they provide the same stimulus for progression in strength training. Furthermore, the lack of ample space also constrained the variety of dynamic movements that can be performed, particularly those requiring large ranges of motion or sustained effort, such as certain forms of aerobic training or plyometric exercises [65]. Incorporating more specific equipment or utilizing outdoor environments (where available) could enhance the program’s ability to provide a wider range of activities and better engage the aerobic system. Future iterations of the program may also consider incorporating a broader spectrum of moderate to high-intensity aerobic exercises that can be performed within home settings or with minimal equipment, allowing for a more comprehensive approach to fitness [66]. Moreover, while this study shows that alternative equipment can be adapted for use, it is essential to recognize that traditional gym-based equipment and broader spaces are more effective in delivering varied and progressive workouts at higher intensities [67,68].

Integrating our training program into remote training platforms presents a flexible approach for users. This could involve direct engagement with an instructor, or alternatively, it could be delivered as a virtual sparring partner. In such a scenario, using technology, such as Inertial Measurement Units, would be essential to facilitate real-time feedback on executing motor tasks [69,70,71]. This feedback mechanism is crucial for ensuring the correct performance of exercises, thereby enhancing the overall efficacy of the training experience. Incorporating advanced technology not only aids in abilities acquisition but also contributes to the safety and involvement effectiveness of remote training [72,73,74]. Additionally, incorporating neurofeedback devices could provide insights into cognitive feedback, allowing individuals to monitor their mental engagement and stress levels [75,76,77]. Aligning these technologies with our training programs can create a comprehensive solution suitable for scenarios like the pandemic or other situations where participants cannot leave their homes. This synergy between technology, artificial intelligence, machine learning and tailored exercise programming may improve physical outcomes and support mental well-being by enabling continuous monitoring and adaptive training, making remote fitness more effective and accessible [35].

## 6. Conclusions

Although designed for the specific context of the lockdown, the program could be adapted to reach broader populations or applied in different contexts. For example, using digital tools and minimal equipment makes it easily accessible to individuals in remote areas, workplace environments, and healthcare settings. Additionally, tiered programs tailored to various fitness levels, real-time digital supervision, and community-building elements, such as group challenges or local leaderboards, could help maintain motivation and engagement beyond lockdown scenarios. Furthermore, the program could also be relevant for athletes traveling globally, as it can be easily scaled to accommodate their fitness needs. In conclusion, the 100-days program achieved an important outcome, such as no injuries and no dropouts. Results suggest that distance training, even without specific equipment, can offer physical and motivational benefits, particularly valuable in prolonged isolation. Such programs could be integrated into public health strategies to support the population in emergencies, promote an active lifestyle, and prevent non-communicable diseases. Nevertheless, the study is not without its limitations. The limited number of participants in this study arises from the unprecedented conditions of the COVID-19 pandemic, which hindered the recruitment of a more extensive participant base. Nonetheless, the insights gained are significant, providing foundational evidence regarding the potential of such interventions in unique and constrained circumstances. Future research should aim to validate these findings with larger, more diverse cohorts while also exploring additional factors, such as access to resources, program duration, and social dynamics.

## Figures and Tables

**Table 1:** Anthropometric measurements of participants divided by gender. NV = Not value; values that do not need a second measurement (Age-Height). Participants’ test results comparison in the challenge modality (C), divided by gender. PUp = Push-Up; R_Crunch_ = Reapeated Crunch; SJ = Squat Jump; QFS = Quick Fit Squatting. * = Statistically significant value.

Female	T0	T1	${\mathrm{Diff}\%}_{T0-T1}$		Male	T0	T1	${\mathrm{Diff}\%}_{T0-T1}$
Age (y)	45.5 ± 11.96	NV	NV		Age (y)	44.9 ± 13.55	NV	NV
Height (cm)	1.65 ± 0.04	NV	NV		Height (cm)	176 ± 7.9	NV	NV
BM (kg)	61.9 ± 7.89	61.1 ± 8.56	−1.4		BM (kg)	80.3 ± 11.40	79.0 ± 10.68	−1.5 *
BMI (kg/m^2^) T0	22.6 ± 2.08	22.3 ± 2.35	−1.4		BMI (kg/m^2^) T0	25.2 ± 3.28	24.8 ± 3.10	−1.5 *
Arm (cm)	27.4 ± 1.86	28.3 ± 1.37	3.6		Arm (cm)	30.1 ± 1.83	30.7 ± 2.63	−0.5
Forearm (cm)	23.3 ± 1.78	24.1 ± 1.99	4.0		Forearm (cm)	28.1 ± 2.48	26.1 ± 3.75	−6.2
Abdomen (cm)	80.0 ± 8.25	81.5 ± 8.26	2.6		Abdomen (cm)	88.4 ± 7.72	85.9 ± 9.97	−2.4

**Table 2:** The protocol involved 10 exercise stations, each lasting 1 min. Each station was repeated for three sets, with a 3-min recovery between stations. Before the start of the training session, there was a 5-min warm-up. At the end of the session, 5 min were spent stretching to promote muscle recovery.

	2 DAY	2 DAY	2 DAY	1 DAY
STATION	POWER	ENDURANCE	COORDINATION AND MOBILITY	POSTURAL
1	Push-Up	Jumping Jack	Tree Pose	Bridge
2	Weighted Squat	Mountain Climber	Alternate Monopodalic Push Press	Back Scratch
3	Weighted Rower	Skip	Calf Raises	Leg Raises
4	Dips	Squat Jump	Hip Raises	Plank
5	Step Up	Trunk Extension	Dead Bug	Crunches
6	Plank Twist	Spiderman Mountain Climber	Isometric Superman Plank	Lateral Plank
7	Lying Side Hip Raises	Monopodalic Jump	Superman	Wall Angels
8	Hip Raises	Wall Sit	Bird Dog	Isometric Bridge
9	Superman Plank	Plank	Crunches	Wall Sit
10	Shoulder Raises	Star	Plank	Static Plank

**Table 3:** Mean and standard deviation (SD) of the initial (T0) and final (T1) measurements from the evaluation test, along with the percentage difference (Diff%) between the two measurements, divided by gender. S&R = Sit and Reach; RTR: Ruler Test Right; RTL: Ruler Test Left; STR = Stork Test Right; STL = Stork Test Left; Squat Jump = SJ; Push-Up = PUp; RI = Ruffier Index. * = statistically significant value.

Female	T0	T1	${\mathrm{Diff}\%}_{T0-T1}$		Male	T0	T1	${\mathrm{Diff}\%}_{T0-T1}$
V-S&R (cm)	12.5 ± 4.51	14.8 ± 6.65	20 *		V-S&R (cm)	4.4 ± 6.33	8.8 ± 10.69	69 *
RTR (cm)	18.1 ± 11.37	19.5 ± 19.12	12.1		RTR (cm)	33.6 ± 27.34	34.7 ± 30.09	0.4
RTL (cm)	10.5 ± 4.20	10.7 ± 5.95	17.6		RTL (cm)	23.7 ± 24.68	22.4 ± 22.86	−7.7
STR (s)	9.0 ± 6.06	5.0 ± 4.67	−22 *		STR (s)	8.1 ± 7.30	3.6 ± 1.70	−45
STL (s)	6.3 ± 4.65	6.7 ± 5.76	−24 *		STL (s)	9.4 ± 9.34	3.8 ± 1.91	−45
SJ (cm)	22.8 ± 5.62	23.0 ± 6.68	11		SJ (cm)	20.7 ± 13.05	28.2 ± 12.40	159
PUp (rep)	32.6 ± 4.36	30.7 ± 11.41	−8 *		PUp (rep)	37.6 ± 17.91	38.2 ± 16.73	5
Ruffier (au)	6.5 ± 2.14	9.8 ± 2.90	65		Ruffier (au)	7.7 ± 4.41	8.5 ± 4.61	24

**Table 4:** Mean and standard deviation (SD) of the initial (T0), intermediate (T1), and final (T2) phases of the PUCRUSQUA, along with the percentage differences (Diff%) between the measurements, divided by gender. R_Push-Up_: Repeated Push-Up; R_Crunch_: Repeated Crunch; R_Squat_: Repeated Squat; QFS = Quick Fit Squatting. * = statistically significant value.

		T0	T1	T2	${\mathrm{Diff}\%}_{T0-T1}$	${\mathrm{Diff}\%}_{T1-T2}$	${\mathrm{Diff}\%}_{T0-T2}$
Female	R_Push-Up_ (rep)	31.8 ± 4.19	37.5 ± 12.69	44.8 ± 11.70	17 *	24	43 *
	R_Crunch_ (rep)	71.5 ± 30.40	81.0 ± 24.94	60.8 ± 14.41	18	−20	-6
	R_Squat_ (rep)	126.0 ± 17.80	139.5 ± 16.09	147.5 ± 27.45	11 *	5	17 *
	QFS (s)	150 ± 21.6	180.0 ± 0.0	180.0 ± 0.0	22 *	5	17 *
Male	R_Push-Up_ (rep)	28.3 ± 12.09	32.9 ± 9.53	38.8 ± 10.86	23 *	23	52 *
	R_Crunch_ (rep)	62.0 ± 44.28	90.9 ± 47.71	92.1 ± 51.72	71 *	4	64 *
	R_Squat_ (rep)	107.4 ± 36.31	129.8 ± 42.82	122.0 ± 41.90	23 *	−6	14 *
	QFS (s)	153 ± 26.9	180.0 ± 0.0	180.0 ± 0.0	21 *	−6	14 *

## Data Availability

No new data were created or analyzed in this study. Data sharing is not applicable to this article.
